# Selected Cognitive Abilities in Elite Youth Soccer Players

**DOI:** 10.1515/hukin-2015-0129

**Published:** 2015-12-30

**Authors:** Veronika Baláková, Petr Boschek, Lucie Skalíková

**Affiliations:** 1 Department of Pedagogy, Psychology and Didactics, Faculty of Physical Education and Sport, Charles University in Prague; 2 Department of Psychology, Faculty of Arts, Charles University in Prague; 3 Analyse Group, s.r.o

**Keywords:** talent identification, Vienna Test System, soccer, cognitive abilities

## Abstract

The identification of talent in soccer is critical to various programs. Although many research findings have been presented, there have been only a few attempts to assess their validity. The aim of this study was to determine the relationship between talent and achievement variables in the Vienna Test System. The participants were 91 Czech soccer players, representing four youth soccer teams, who were born in the year 2000. These boys were divided into two groups according to their coaches’ assessments using a TALENT questionnaire. A two-factor model (component 1: “kinetic finesse”; component 2: “mental strength”) was designed to interpret the responses of the coaches on the questionnaire. The Vienna Test System was used to determine the level of players’ cognitive abilities. In total, the subjects performed seven tests in the following order: Raven’s Standard Progressive Matrices (SPM), a reaction test (RT), a determination test (DT), a visual pursuit test (LVT), a Corsi Block-Tapping Test (CORSI), a time/movement anticipation test (ZBA), and a peripheral perception test (PP). To analyze the relationship between talent and achievement variables within the Vienna Test System, correlation analyses were performed. The results revealed that the talented group attained significantly better results on only 1 of the 16 variables, which was ZBA2: movement anticipation - deviation of movement median (r = .217, p = .019). A comparison of the two talent components showed that component 1 (“kinetic finesse”) was a more significant factor than component 2 (“mental strength”). Although we observed statistically significant correlations, their actual significance remains questionable; thus, further research is required.

## Introduction

Talent in soccer is identified using various programs, and their application is currently increasing at professional soccer clubs and national associations worldwide.

Future success in soccer depends on a host of internal factors, including personality type, body type, speed, and endurance, as well as various external variables, such as the nature of coaching, opportunities to practice, and social and cultural factors.

Characteristic identification schemes that differentiate skilled from less-skilled performers, along with the role of heredity and environment in the development of expertise, have been reviewed in several articles (e.g., Règnier et al. (1993) and Williams and Reilly (2000)).

According to Janelle and Hillman (2003), athletes must excel in no less than four domains: physiological, technical, cognitive (tactical/strategic; perceptual/decision-making), and emotional (regulation/coping; psychological). Cognitive expertise can be divided into two subdomains: tactical knowledge, which involves the ability to determine not only what strategy is most appropriate in a given situation but also whether the strategy can be successfully executed, and perceptual skills, which consist of pattern recognition, anticipatory cue extraction and use, visual search strategies and signal detection. Williams (2000) and Vickers (2007) similarly stated that athletes should be able to anticipate what is most important in the environments in which they play. Moreover, athletes are expected to attend to critical cues, concentrate at the appropriate moments, retrieve necessary information from memory at the proper time, solve problems when they arise, and, ultimately, make a correct decision under time constraints. Similar factors (sensorimotor abilities) and other factors (intellectual, creative and socioaffective abilities) are included in the Differentiated Model of Giftedness and Talent (DMGT) by Gagné and Rossum (in Vaeyens et al., 2008).

The structure of these assumptions for good performance is not addressed, only the concepts in general, and their instantiation within particular sports, especially soccer, is unclear.

The ability to anticipate and make decisions is presumed to be important in soccer, particularly at the elite level (Reilly and Williams, 2000; Reilly et al., 2000; Williams et al., 2000).

Based on their research regarding the factor structure of the technical and coordination potential of soccer players, Witkowski et al. (2006) emphasized spatial orientation, speed of response, and sense of movement rhythm as the principal coordination abilities.

Ljach et al. (2012) measured the professionally important sensorimotor cognitive abilities (PISMCAs) of young soccer players and examined how these abilities could be improved. The results showed that experimental groups that underwent special training programs demonstrated significant improvement compared to control groups.

Even though many research findings have been presented (the total number of articles in the Journal of Sports Sciences, Volume 18, Issue 9, 2000) and a few models of talent identification have been generated, only cursory attempts have been made to assess their validity. This study addresses this knowledge gap.

The aim of this study was to determine whether talented players in sports, specifically soccer, could be identified based on their psychological characteristics (sensorimotor and psychomotor). We chose the Vienna Test System (VTS), as the authors had previously used this battery to identify talented young athletes. We chose subtests that we assumed measured abilities that were determinants of success (reaction time, motor time, visual memory, peripheral perception, etc.). The battery was complemented by a simple nonverbal test of general intelligence to eliminate the impact of the subjects’ intellect on the test results. Therefore, this study was designed to determine the relationship between talent variables and achievement variables within the VTS and to compare general cognitive abilities between talented and less-talented youth soccer players for the purpose of identifying the distinct cognitive characteristics exhibited by talented players.

## Material and Methods

### Participants

The participants were 91 Czech soccer players representing three youth soccer teams in Prague and one youth soccer team in Pilsen (24 players: Viktoria Plzeň; 26 players: Dukla Praha; 22 players: Slavia Praha: and 19 players: Bohemians 1905). All players were males who were born in the year 2000 (they were 13 years old during testing). The teams were recruited based on their performance level (teams ranked among the best in the Czech Republic) and coaches’ availability and interest. The subjects were divided into two groups according to their coaches’ assessments using a TALENT questionnaire.

Informed consent from the children and their parents, along with approval from the Charles University in Prague ethics committee, was obtained.

### Measures

#### Phase 1

For the purpose of talent characteristic identification, a standard method of expert assessment was used. Coaches with a UEFA A, UEFA B, or Youth coach UEFA A license (N = 40) completed a list of open questions regarding personal determinants for preparing and identifying a modern soccer player. Their most frequent answers were used to produce the TALENT questionnaire.

The TALENT questionnaire consisted of one overall question about talent in general and 14 statements focused on 3 talent categories – movement, technique, and psychological characteristics. Responses were recorded on a 6-point Likert scale ranging from *Not typical at all* (1) to *Very typical* (6). The aim of this tool was to divide the players into two groups – talented and less talented - and to derive a description of the players’ giftedness. The first overall question was instrumental in distributing the players into two groups, and the remaining 14 questions were used to obtain more objective assessments from the coaches such that the structure of the talent construct could be shown.

The proposed TALENT questionnaire covered 3 domains (movement, technique, psychological characteristics). Based on 3-factor and 2-factor component analyses, in contrast to the originally hypothesized 3-factor model, a 2-factor model better fitted the results from this questionnaire.

The structure, or dimensionality, of the TALENT questionnaire was analyzed based on an exploratory two-factor principal component analysis (PCA), followed by orthogonal rotation “VARIMAX” and oblique rotations “OBLIMIN”. The reason for the orthogonal rotation “VARIMAX” application was the assumption that the 2 components were not correlated. As shown in Table 1, some items showed cross-loading. Therefore, oblique rotation “OBLIMIN” was used considering the assumption that the 2 components strongly correlated. In this “OBLIMIN” application, there were no cross-loadings, and it was accepted as the final model. These two components explained 63.4% of the total variance. The extracted components represented a very meaningful dimensionality reduction: component 1: “kinetic finesse”; component 2: “mental strength”. Scores in both dimensions were obtained as the unweighted sum of the scores of the relevant items.

The first overall question included in the TALENT questionnaire (completed by the coaches) asked for a dichotic characterization – non-talented, talented (0, 1). The biserial correlations shown in Table 2 revealed that the coaches’ evaluations were “closer” (more similar) to the scores on component 1 than to the scores on component 2. The correlation between the 2 components with regard to the unweighted sums of scores was very similar (.55) to the correlation between the extracted components (.51; Table 1).

#### Phase 2

The VTS (Schufried GmbH, Austria) was used to determine each player’s level of cognitive skill (ability). The study used a computerized version of the VTS, which had been assessed for reliability and validity before this study (Schufried, 2001; Whiteside et al., 2003; Gierczuk and Ljach, 2012). To conduct our testing of players’ cognitive abilities, we selected 7 tests.

The tests and variables were selected based on the components of the VTS (Schufried GmbH, Austria) and the results of a previous research review. VTS recommendations related the following systems to talent detection: the Expert System Sport and the Young Talent and Expert System Sport – Team Sport. These two systems contained tests such as a reaction test (RT), the Corsi Block-Tapping Test (CORSI), a long-term selective attention test (SIGNAL), a visual pursuit test (LVT), a Stroop test (of interference tendency), a visual memory test (VISGED), the Gestalt perception test (GESTA), a time/movement anticipation test (ZBA), and a determination test (DT).

### Procedures

The testing procedures were conducted during a summer training period. The selected VTS tests were performed under standard conditions. Prior to commencing the tests, each subject was familiarized with the test procedures and then participated in an introductory test. The measurements were recorded before a training session or during a day off. Players were not aware of their final distribution into one of the two groups (talented or less talented).

In total, the subjects performed 7 tests over 50 minutes in the following order:

Raven’s Standard Progressive Matrices (SPM – S5 version): this test is used as a non-verbal assessment of general intelligence in people with average capacity on the basis of deductive thinking;
RT (S4 version): this test measures reaction time and motor time in response to simple and complex visual or acoustic signals;
DT (S1 version): this test examines reactive stress tolerance, attention and reaction speed in situations requiring continuous, swift and varying responses to rapidly changing visual and acoustic stimuli;
LVT (S2 version): this test measures visual orientation ability and skill in gaining an overview;
CORSI (S2 version): this test is used for the assessment of visual short-term memory capacity and implicit visuospatial learning;
ZBA (S3 version): this test measures the ability to estimate speed and movement of objects in space; and
Peripheral perception test (PP); this test is designed to assess the perception and processing of peripheral visual information.

The evaluation involved the following variables.

- SPM: total correct answers
- RT: reaction time median, motor reaction time median
- DT: the number of correct reactions, the number of incorrect reactions, the number of omitted reactions
- LVT: the number of items solved correctly within the set time limit, the number of items solved incorrectly, working time
- CORSI: the longest sequence duration
- ZBA: deviation of time median, deviation of movement median
- PP: overall field of vision, left/right visual angle, tracking deviation

### Statistical Analysis

For comparison between talent variables (TALENT 0, 1; Comp 1; and Comp 2) and achievement variables in the VTS, correlation analyses were performed. Because of the ordinal characteristic of the evaluation scale (Comp 1, Comp 2) and considering the characteristics and distribution of these variables, Spearman correlation coefficients (r_s_) were calculated. Point-biserial correlation coefficients (r_pb_) were used for dependence analysis between achievement variables and talent evaluation scores. All analyses were performed using SPSS software.

## Results

The results revealed that the talented group attained significantly better results on only 1 of the 16 variables, which was *ZBA2: Movement anticipation - deviation of movement median*. The talented group exhibited greater mean and median movement anticipation than the less-talented group (Mean 33.6 vs 27.5; Median 33.0 vs 23.5). The comparison of the two talent components showed that component 1 (“kinetic finesse”) was more significantly associated with the TALENT parameter than component 2 (“mental strength”), especially item 6 of 16 in component 1, which included RT2, LVT1, LVT6, ZBA2, PP1, and PP2, in contrast to item 2 of 16 in component 2 (which included LVT5-4 and ZBA2).

## Discussion

Based on recently presented research findings supporting the assumption that cognitive abilities are important for talent identification, 91 youth soccer players were tested using the VTS.

The examination of their coaches’ assessment via the TALENT questionnaire showed that the overall dichotomy of the players was more strongly determined by component 1 (“kinetic finesse”) than by component 2 (“mental strength”). We assume that these visible characteristics (e.g., coordination, movement technique, reaction time) can be more easily perceived and identified by coaches and that coaches primarily use their common sense and their visual experience to recognize patterns of movement among their players (Christensen, 2009). In contrast, psychological/personal characteristics (e.g., self-discipline, effort to exceed, ambition to win) appear to be less noticeable to coaches. Coaches acknowledge the importance of these characteristics in practice (they reported them in the pilot survey); however, they apparently fail to recognize and describe them. This observation may be considered in further research on this topic.

The correlations between VTS variables and talent indicators showed nearly no differences between talented and less-talented youth soccer players. Similarly, Huijgen et al. (2014) examined differences in physiological, technical and tactical characteristics between the selected and deselected soccer players but found only non-significant differences in psychological characteristics between the groups. The talented group attained only one significantly better result on 1 of the 16 evaluated variables, ZBA2: Movement anticipation - deviation of movement median. It could be expected that players who more effectively anticipate ball movement (anticipation of the placement of a ball drop/pass, movement of a teammate/opponent based on their position, etc.) are more likely to score. Such players could be more likely to be assessed by their coaches as talented. One open question is the extent to which these movement anticipation skills and the ability to successfully “read” and anticipate a play are related to the player’s experience. Generally, human perceptual skills improve with experience; i.e., we refine our perceptual strategies by practicing a given situation. Thus, learning processes imply improvements in complex perceptual-based skills as a consequence of training (Sowden et al., 2000; Ste-Marie et al., 2012). The report by Williams (2000) supports the concept that expert players do not have greater visual skills than novice players. Similarly, Roca et al. (2012) discovered that the number of hours spent in soccer-specific play activity during childhood was the strongest predictor of performance on a test of anticipation and decision making. In the future, it could be useful to compare players’ cognitive skills according to their experience and to specifically analyze the number of training sessions/months/years, as other researchers have explored (e.g., Loffing and Hagemann, 2014; Roca et al., 2013; Ryu et al., 2013; Vila-Maldonado et al., 2014).

VTS more effectively predicted talent when utilized from the perspective of component 1 than when considering only TALENT 0, 1 (6 significant results for component 1 vs 1 significant result for TALENT 0, 1). Although we observed statistically significant correlations, their actual significance remains questionable. For instance, the coefficient of determination for the largest correlation coefficient (r = .26 for the correlation between LVT5-4 and component 2) accounts for only approximately 7% of the common variability in these variables. This result did not fit our assumption that there would be several significant correlations between talent variables and VTS achievement variables. However, this result supports the concept that although differences in skills between groups become more evident on tasks directly related to the specific domain, there are no differences in performance on general laboratory tests between groups (Vila-Maldonado et al., 2014; Abernethy et al., 1994). Additionally, other researchers (McPherson, 1994; Unnithan et al., 2012; Vaeyens et al., 2008) have suggested the development of more representative real-world tasks.

Our results could be further discussed based on additional considerations.

The first consideration regards the VTS. There are several questions concerning the validity of this system. Although the VTS was assessed in terms of reliability and validity before this study (Schufried, 2001; Whiteside et al., 2003; Gierczuk and Ljach, 2012), some of the results from our assessments did not correspond to our observations during testing. For example, the DT and the number of omitted answers variable could not indicate that the players exhibited a low level of stress tolerance, as the authors described. Many players omitted certain items but remained calm and good-tempered and continued the testing procedure. Alternatively, other players stopped the test, began to get upset, and chose to cry or give up. Surprisingly, the “upset” players achieved better average results on this variable, which the authors had described as “stress tolerance”. The general and vague designs of individual tests measuring cognitive abilities, as well as the laboratory conditions, result in unreliability in predicting the talent of young soccer players. Thus, the validity and reliability of the VTS should be further investigated.

Furthermore, it is questionable whether the high dimensionality of the talent construct, which contains many determinants, enables the detection of talent in one individual predominantly according to motion determinants and in another individual predominantly according to psychological determinants. This dimensionality of talent suggests that the identification of talent is distinct between individuals and that different methods should be used to evaluate talent. This uniqueness also supports the recommendations of Goldsmith as presented on his website (http://www.wgcoaching.com/talentidtips/).

This hypothesis is further supported by the observation that although all participants were the same age at the time of testing, they had reached different stages of development, and their biological and mental ages could be ahead of or behind their chronological ages. Helsen et al. (2012) addressed the relative age effect, or the asymmetry in birth distribution favoring players who were born earlier rather than later in the selection year. They presented data illustrating that the age effect remained pervasive, but they concluded that its negative impact may be alleviated over time. It has also been suggested that many of the qualities that distinguish top athletic performance in adults may not be apparent until late adolescence (Vaeyens et al., 2008). In addition, according to Gonçalves et al. (2012), talent identification is a long process, and the earlier a decision is made, the greater the uncertainty of the outcome. Similarly, Vaeyens et al. (2008) summarized that talent identification and development programs should be dynamic and interconnected and should consider maturity status and the potential to develop rather than excluding children at an early age. Given the young age of the participants, we can assume that the stage at which their talent is revealed could vary.

Coaches also remarked in hindsight that they would have evaluated their players differently using the TALENT questionnaire. Thus, it is evident that talent identification research requires longitudinal monitoring, and measurements must include all variables, as growth acceleration and gained muscle mass could be misleading characteristics. This multidisciplinary perspective is encouraged by many researchers (Christensen, 2009; Gonaus and Müller, 2012; Vandendriessche et al., 2012).

Our conclusion may be consistent with a previous review, which reported that efforts to measure talent using objective tests of basic cognitive and perceptual motor abilities could be remarkably unsuccessful in predicting final performance in specific domains (Ericsson et al., 1993).

Despite the present trend in selecting young players based on multifactorial attributes, in this study, we tested only cognitive abilities. Although our results did not validate our hypothesis, they indicated several future recommendations, such as including additional observers for completion of the talent questionnaire, teaching coaches to use/“observe” psychological characteristics, considering players’ experience, using a soccer-related testing battery or system, and, finally, performing multiyear case studies to develop additional unique perspectives.

## Figures and Tables

**Figure 1:**
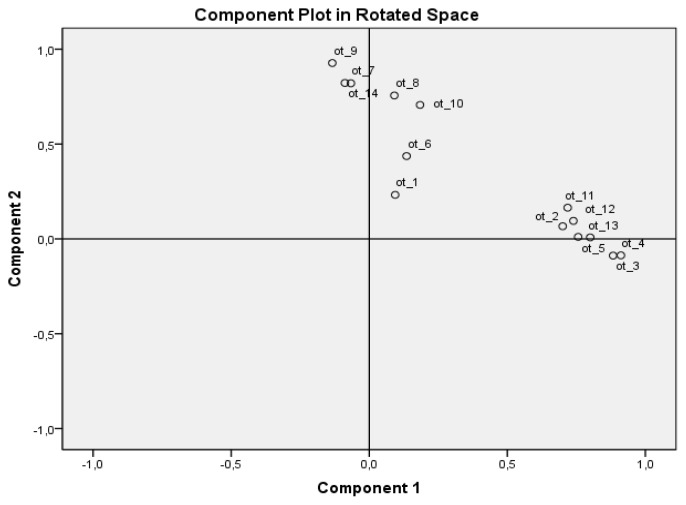
Placement of items in a two-dimensional component space when oblique rotation (OBLIMIN) was applied Component 1 – component “kinetic finesse”, Component 2 – component “mental strength”

**Table 1:** Rotated component loadings

Rotated Component Matrix	VARIMAX		OBLIMIN	

	Comp 1	Comp 2	Comp 1	Comp 2
it_4	.855		.911	
it_3	.827		.883	
it_13	.773		.800	
it_12	.737	.290	.739	
it_11	.736	.351	.719	
it_5	.732		.757	
it_2	.692	.252	.701	
it_9		.858		.927
it_7		.773		.820
it_14		.769		.822
it_8	.287	.754		.756
it_10	.364	.731		.706
it_6		.457		.436
it_1		.253		.232

Suppressed factor loadings < .25; correlation between components with regard to oblique rotations

OBLIMIN: r = .51; Comp 1 – component “kinetic finesse”, Comp 2 – component “mental strength”

**Table 2:** Correlations between extracted components and talent indicators

	Correlation		Correlation		Correlation	
	
	TAL		Comp 1		Comp 2	
	
	r =	p =	r =	p =	r =	p =
Talent_01	1		.562 a	.000	.425 a	.000
Comp 1	.562^a^	.000	1		.554 b	.000
Comp 2	.425 a	.000	.554^b^	.000	1	

a Biserial correlation;

b Pearson correlation

**Table 3:** Correlations between VTS variables, talent indicators and extracted components

	Biserial r_pb_		Spearman r_s_		Spearman r_s_	
	
	TALENT		Comp 1		Comp 2	
	
Main variables	r =	p =	r =	p =	r =	p =
SPM	−.004	.484	.122	.125	.031	.386
RT1	−.034	.376	−.091	.197	−.151	.076
RT2	−.100	.173	.239	.011	−.034	.376
DT1	.009	.467	−.002	.494	.127	.114
DT2	.094	.188	.145	.085	.032	.383
DT3	.129	.112	.135	.101	−.006	.477
LVT1	−.014	.448	−.195	.032	−.168	.056
LVT5-4	.110	.149	.164	.060	.260	.006
LVT6	.101	.171	.236	.012	−.020	.426
CORSI	.104	.163	.004	.486	−.031	.385
ZBA1	−.029	.393	−.022	.418	−.005	.481
ZBA2	.217	.019	.205	.026	.253	.008
PP1	−.067	.263	−.179	.045	.043	.343
PP2	−.071	.251	−.215	.020	.056	.298
PP3	−.046	.332	−.129	.111	.050	.318
PP4	−.138	.096	−.064	.273	−.159	.066

SPM: total correct answers; RT1: reaction time median; RT2: motor reaction time median; DT1: number of correct reactions; DT2: number of incorrect reactions; DT3: number of omitted reactions; LVT1: number of items solved correctly within the set time limit; LVT5-4: number of items solved incorrectly; LVT6: working time; CORSI: the longest sequence length; ZBA1: deviation of time median; ZBA2: deviation of movement median; PP1: overall field of vision; PP2: left visual angle; PP3: right visual angle; PP4: tracking deviation.
